# Supplementary material for: Modeling glioblastoma heterogeneity as a dynamic network of cell states
Source: Mol Syst Biol. 2021 Sep 16;17(9):e10105. doi: 10.15252/msb.202010105 (PMC8444284; doi:10.15252/msb.202010105)
Supplement: Supplementary file 5 — Source Data for Figure 3 [file MSB-17-e10105-s001.zip › Figure3A_sourcedata/GSEA_3065/hallmarks_state5.GseaPreranked.1623416302988/gsea_report_for_na_neg_1623416302988.html]

Report for na\_neg 1623416302988 [GSEA]

| GS  follow link to MSigDB | GS DETAILS | SIZE | ES | NES | NOM p-val | FDR q-val | FWER p-val | RANK AT MAX | LEADING EDGE || 1 | HALLMARK\_OXIDATIVE\_PHOSPHORYLATION | Details ... | 187 | -0.72 | -2.70 | 0.000 | 0.000 | 0.000 | 1564 | tags=64%, list=17%, signal=76% |
| 2 | HALLMARK\_MYC\_TARGETS\_V1 | Details ... | 194 | -0.71 | -2.61 | 0.000 | 0.000 | 0.000 | 1636 | tags=63%, list=18%, signal=75% |
| 3 | HALLMARK\_REACTIVE\_OXYGEN\_SPECIES\_PATHWAY | Details ... | 37 | -0.64 | -1.89 | 0.000 | 0.000 | 0.001 | 402 | tags=32%, list=4%, signal=34% |
| 4 | HALLMARK\_DNA\_REPAIR | Details ... | 127 | -0.52 | -1.84 | 0.000 | 0.000 | 0.001 | 1169 | tags=33%, list=13%, signal=37% |
| 5 | HALLMARK\_PI3K\_AKT\_MTOR\_SIGNALING | Details ... | 82 | -0.50 | -1.67 | 0.000 | 0.012 | 0.073 | 1203 | tags=27%, list=13%, signal=31% |
| 6 | HALLMARK\_FATTY\_ACID\_METABOLISM | Details ... | 105 | -0.48 | -1.64 | 0.001 | 0.013 | 0.092 | 2150 | tags=44%, list=24%, signal=57% |
| 7 | HALLMARK\_MYC\_TARGETS\_V2 | Details ... | 47 | -0.52 | -1.62 | 0.006 | 0.014 | 0.119 | 2724 | tags=51%, list=30%, signal=73% |
| 8 | HALLMARK\_E2F\_TARGETS | Details ... | 187 | -0.41 | -1.52 | 0.001 | 0.042 | 0.342 | 1636 | tags=28%, list=18%, signal=34% |
| 9 | HALLMARK\_ADIPOGENESIS | Details ... | 153 | -0.41 | -1.47 | 0.006 | 0.058 | 0.488 | 1789 | tags=36%, list=20%, signal=44% |
| 10 | HALLMARK\_PEROXISOME | Details ... | 66 | -0.44 | -1.44 | 0.029 | 0.073 | 0.629 | 858 | tags=21%, list=10%, signal=23% |
| 11 | HALLMARK\_G2M\_CHECKPOINT | Details ... | 180 | -0.38 | -1.37 | 0.014 | 0.123 | 0.834 | 2469 | tags=35%, list=27%, signal=47% |
| 12 | HALLMARK\_TGF\_BETA\_SIGNALING | Details ... | 45 | -0.45 | -1.36 | 0.073 | 0.125 | 0.855 | 2327 | tags=36%, list=26%, signal=48% |
| 13 | HALLMARK\_P53\_PATHWAY | Details ... | 134 | -0.33 | -1.19 | 0.174 | 0.410 | 1.000 | 1353 | tags=19%, list=15%, signal=22% |
| 14 | HALLMARK\_ALLOGRAFT\_REJECTION | Details ... | 61 | -0.37 | -1.18 | 0.181 | 0.393 | 1.000 | 518 | tags=15%, list=6%, signal=16% |
| 15 | HALLMARK\_GLYCOLYSIS | Details ... | 140 | -0.31 | -1.11 | 0.260 | 0.564 | 1.000 | 1050 | tags=17%, list=12%, signal=19% |
| 16 | HALLMARK\_MTORC1\_SIGNALING | Details ... | 173 | -0.29 | -1.08 | 0.323 | 0.615 | 1.000 | 1789 | tags=34%, list=20%, signal=41% |
| 17 | HALLMARK\_HYPOXIA | Details ... | 124 | -0.29 | -1.04 | 0.375 | 0.685 | 1.000 | 1133 | tags=17%, list=13%, signal=19% |
| 18 | HALLMARK\_APOPTOSIS | Details ... | 109 | -0.29 | -1.02 | 0.431 | 0.726 | 1.000 | 1411 | tags=24%, list=16%, signal=28% |
| 19 | HALLMARK\_APICAL\_JUNCTION | Details ... | 114 | -0.28 | -0.99 | 0.498 | 0.771 | 1.000 | 1144 | tags=16%, list=13%, signal=18% |
| 20 | HALLMARK\_ESTROGEN\_RESPONSE\_LATE | Details ... | 103 | -0.27 | -0.92 | 0.632 | 0.924 | 1.000 | 1660 | tags=26%, list=18%, signal=32% |
| 21 | HALLMARK\_UNFOLDED\_PROTEIN\_RESPONSE |  | 98 | -0.27 | -0.92 | 0.623 | 0.887 | 1.000 | 1801 | tags=29%, list=20%, signal=35% |
| 22 | HALLMARK\_HEME\_METABOLISM |  | 120 | -0.25 | -0.89 | 0.702 | 0.920 | 1.000 | 2105 | tags=28%, list=23%, signal=37% |
| 23 | HALLMARK\_UV\_RESPONSE\_UP |  | 111 | -0.25 | -0.88 | 0.698 | 0.918 | 1.000 | 2118 | tags=31%, list=24%, signal=40% |
| 24 | HALLMARK\_TNFA\_SIGNALING\_VIA\_NFKB |  | 104 | -0.25 | -0.87 | 0.746 | 0.905 | 1.000 | 2373 | tags=34%, list=26%, signal=45% |
| 25 | HALLMARK\_XENOBIOTIC\_METABOLISM |  | 95 | -0.25 | -0.85 | 0.756 | 0.903 | 1.000 | 1895 | tags=28%, list=21%, signal=36% |
| 26 | HALLMARK\_INTERFERON\_GAMMA\_RESPONSE |  | 95 | -0.24 | -0.80 | 0.863 | 0.965 | 1.000 | 2198 | tags=27%, list=24%, signal=36% |
| 27 | HALLMARK\_NOTCH\_SIGNALING |  | 22 | -0.29 | -0.77 | 0.777 | 0.975 | 1.000 | 1050 | tags=18%, list=12%, signal=21% |
| 28 | HALLMARK\_MITOTIC\_SPINDLE |  | 175 | -0.21 | -0.76 | 0.939 | 0.956 | 1.000 | 2229 | tags=23%, list=25%, signal=31% |
| 29 | HALLMARK\_SPERMATOGENESIS |  | 57 | -0.21 | -0.68 | 0.947 | 1.000 | 1.000 | 2056 | tags=21%, list=23%, signal=27% |
| 30 | HALLMARK\_INTERFERON\_ALPHA\_RESPONSE |  | 56 | -0.19 | -0.60 | 0.983 | 0.990 | 1.000 | 1101 | tags=14%, list=12%, signal=16% |
Table: Gene sets enriched in phenotype **na **[plain text format]****
